# Expression of Axl Receptor Tyrosine Kinase and Its Association With Ki-67 Proliferation Marker, BCL-2 Anti-apoptotic Protein, Hormone Receptor Status, and HER2/Neu Status in Breast Cancer Among Women From Duhok, Iraq

**DOI:** 10.7759/cureus.70204

**Published:** 2024-09-25

**Authors:** Sada S Mohammed, Hanaa Al Mahmoodi, Mayada I Yalda

**Affiliations:** 1 Department of Molecular and Medical Biotechnology, College of Biotechnology, Al-Nahrain University, Baghdad, IRQ; 2 Department of Pathology, College of Medicine, University of Duhok, Duhok, IRQ

**Keywords:** axl receptor, bcl-2 anti-apoptotic protein, breast cancer, er, her2/neu, ki-67 proliferation marker, pr

## Abstract

Background

Breast cancer (BC) is the most prevalent cancer among women worldwide, contributing to high mortality rates, especially in Iraqi women. Detecting the disease before metastasis may increase survival chances for many patients, but that is not the case for most of them. Thus the search for new prognostic biomarkers or testing the relevance of existing ones could contribute to therapeutic decisions complementing the traditional methods, including TNM (tumor, node, and metastasis) staging, tumor grade, and other clinicopathological features in addition to the use of estrogen receptor (ER), progesterone receptor (PR), and human epidermal growth factor receptor 2 (HER2/neu). The Axl receptor is frequently associated with invasion, migration, poor prognosis, and angiogenesis. Furthermore, its association with chemotherapy and targeted therapy resistance makes it an ideal biomarker for therapeutic targeting.

Methodology

This study involved 50 malignant cases with 25 benign fibroadenoma and non-neoplastic cases represented by inflammatory conditions, collected with their corresponding data from the central lab in Duhok Governorate, Iraq. Expression of Kiel 67 (Ki-67) proliferation marker and B-cell lymphoma 2 (BCL-2) anti-apoptotic protein was measured using immunohistochemistry (IHC) to estimate tumor growth and apoptosis. Gene expression of the Axl receptor was evaluated using reverse transcription-quantitative polymerase chain reaction (RT-qPCR).

Results

Cases with high Ki-67 accounted for 68% and low Ki-67 cases were 32% across the graded groups and were significantly associated with tumor grade, PR, and HER2. BCL-2-negative cases accounted for 62% and BCL-2-positive cases were 38%. It was revealed that BCL-2 had a strong correlation with age, especially in those under 50 years. As for the Axl gene expression, the average fold change in expression in the high-grade (H.) group was 1.74 times higher than in the control group, while in the low/intermediate (L.) group, it was 3.74 times higher. Additionally, when comparing these results with other variables, no significant associations were observed.

Conclusion

Axl receptor was not associated with all of the clinicopathological variables, the expression values were high in malignant tumors in comparison with the benign tumors, and it was found that Axl receptor expression was associated with low/intermediate grade, which is considered a favorable prognostic factor. Although Axl receptor expression was previously linked with proliferation and invasiveness in BC, its association with the Ki-67 proliferation marker and BCL-2 anti-apoptotic protein was not observed.

## Introduction

In 2020, the number of new breast cancer (BC) cases was 2.3 million, and 685,000 deaths were caused by BC worldwide [1]. Between 2000 and 2019, a total of 72,022 BC cases were identified in Iraq among women [2]. A study conducted on BC cases retrieved from the Pathology Department of Duhok Central Laboratory in the timeframe before 2013 suggested a very high mortality rate of 70% in BC cases in Duhok, which showed differences in comparison to other parts of the world [3]. Metastasis is the leading cause of high mortality rates in most cancers. Predicting BC at earlier stages, before metastasis occurs, can significantly increase survival rates. However, BC is often diagnosed after metastasis has already manifested [4]. The most commonly used biomarkers are estrogen receptor (ER), progesterone receptor (PR), and human epidermal growth factor receptor 2 (HER2/neu), which help in treatment planning for BC patients by clinical oncologists. The improved understanding of the molecular characterization of BC and the debut of novel targeted therapies have led to a greater need for potential biomarkers [5]. The progress in the field of translational omics has led to the emergence of refined classifiers at the DNA, RNA, and protein levels to interpret the heterogeneity of BC in a clinically applicable way. The omics knowledge is further expanding biological understanding of BC and paving the way for more opportunities to customize therapy [6]. The Axl receptor tyrosine kinase was first identified in patients diagnosed with “chronic myelogenous leukemia” [7]. This receptor belongs to the TAM (Tyro3, Axl, and Mer) receptor tyrosine kinase subfamily. Axl activates upon binding with growth arrest-specific 6 (Gas6) and serial events occur when the signals are transported from the extracellular matrix into the cytoplasm. Axl receptor is involved with cellular growth, aggregation, migration, and anti-inflammation [8]. In the context of BC, it was concluded that Axl's high expression correlated with poor differentiation, indicating its role in developing BC; additionally, it was revealed that Axl causes the upregulation of ER and PR, but the exact mechanism is not clear and requires further investigation [9]. It was concluded in several studies that the Axl receptor was primarily responsible for forming metastasis in epithelial-like cancers (ER+, HER2+). In triple-negative BC (TNBC), the Axl receptor plays a role in forming primary tumors and metastasis, especially in mesenchymal-like primary tumors [10].

The proliferation marker Ki-67 is used by surgical pathologists. It represents a nuclear-localized immunohistochemistry (IHC) marker that indicates that the cells are proliferating in G1 to S-phase. In tumor samples, the growth fraction is estimated by the Ki-67 index (the percentage of tumor cells that are Ki-67 positive) [11]. In BC, Ki-67 is traditionally used in combination with hormone receptor (HR) status and HER2/neu status to provide an IHC4 score used alongside clinical prognostication tools to identify patients who require cytotoxic chemotherapy in addition to endocrine therapy [12], and its expression is becoming increasingly essential to refrain from over- or undertreatment in respect to the other traditional factors such as TNM (tumor, node, and metastasis) staging, ER/PR+, and HER2/neu- of early BC [13]. The regulator of apoptosis, BCL-2, creates an integral part of the outer membrane of the mitochondria. It was found that BCL-2 expression, especially in the case of BCL-2 translocation to immunoglobulin heavy chain locus, might be the causative of “follicular lymphoma” [14]. It was concluded that BCL-2 expression was associated with HR status and favorable clinicopathologic characteristics while lower BCL-2 expression was connected with unwanted clinical outcomes in BC [15].

High expression of the Axl receptor in BC is primarily involved with invasion, migration, and cellular proliferation. On the other hand, Ki-67 is commonly used as an indicator for cellular proliferation, especially in BC routine testing, while BCL-2 is an indicator for programmed cell death inhibition. This study aimed to identify the relationship of these markers and to comprehensively analyze their association with classical prognostic factors and provide data of significant value to other important prognostic indicators such as pathological grading, tumor size, and axillary lymph node (LN) involvement focusing mainly on Axl receptor expression to comprehend its relationship with the other variables to identify its role as a potential biomarker for therapeutics and diagnosis.

## Materials and methods

Patients, study samples, and ethics

A total of 75 formalin-fixed paraffin-embedded (FFPE) tissue samples were arranged in groups: H. group (n = 25), represented by high-grade malignant tumors; L. group (n = 25), represented by low/intermediate grade malignant tumors; and C. group (n = 25), represented by fibroadenoma benign tumors for comparison and non-neoplastic cases represented by inflammatory conditions as a control group.

The inclusion criteria for tissue samples were as follows: cases with histopathological sheets containing all the required clinicopathological data, BC biopsies available as FFPE tissue blocks, biopsies including the entire tumor surrounded by uninvolved breast tissue, and biopsies taken with LNs. The exclusion criteria included cases with missing information, cases with missing FFPE blocks, small biopsies taken by true-cut biopsy only, since they contain no information about the LN involvement or the surrounding breast tissue, and biopsies taken without LNs. The tissue block ages ranged from two months to six years (from 2018 to 2024).

BC sample cases were collected along with their corresponding clinicopathological data, including HR status and HER2/neu status, from the central laboratory in Duhok Governorate, Iraq. Ethical approval was obtained from the Science Research Ethics Committee at Al-Nahrain University, College of Biotechnology (approval number: 2; dated: December 23, 2023). Tissue blocks were gathered with their corresponding hematoxylin and eosin (H&E) slides; however, the majority of the slides were unavailable. Therefore, H&E staining was performed for the missing cases. A pathologist reviewed all H&E slides to confirm the original diagnosis and to identify the malignant regions of interest for total RNA extraction.

Immunohistochemistry for Ki-67 and BCL-2

For each tissue block, four sections with 4 µm thickness were sectioned and mounted on two charged adhesion microscope slides using Automated Rotary Microtome (Leica, Wetzlar, Germany) and water bath at 50-54°C (Charan Associates, Bangkok, Thailand).

The slides’ baking step was performed by placing the slides in a vertical position in the hot air oven at 65°C overnight, followed by dewaxing and rehydration steps achieved by twice immersing in xylene for 15 minutes each, twice immersing in absolute ethanol for five minutes, 95% ethanol for five minutes, 90% ethanol for five minutes, 80% ethanol for five minutes, and 70% ethanol for five minutes, and finally submerging in distilled water for five minutes. The antigen retrieving step was accomplished by immersing in Tris-EDTA (ethylenediaminetetraacetic acid) buffer with pH 9 (Scharlau, Barcelona, Spain) in a water bath at 100°C for 20 minutes, and then cooling down at room temperature followed by washing.

An indirect IHC with a polymer-based detection method was employed by utilizing a master polymer plus detection system (peroxidase) (Master Diagnostica, Granada, Spain). Primary antibodies, including rabbit anti-human Ki-67 monoclonal antibody (clone SP6) and rabbit anti-human BCL-2 monoclonal antibody (clone EP36) from Master Diagnostica, were used. The staining procedures were applied according to the manufacturer’s protocols. Positive controls for both markers were obtained according to the primary antibodies' manufacturer. For BCL2 and Ki-67 monoclonal antibodies, one tissue block of normal tonsil was used, and for negative control, all reagents except the primary antibody were applied (positive staining indicates a lack of specificity of the antibody). Stained slides were examined using a light microscope by a specialist pathologist. For Ki-67 expression, initially, the whole slide was examined microscopically on low power to identify tumor "hot spots," and then the microscope was focused on the 20x objective, and 300 tumor cells were counted to identify the percentages of positive cells in each case. For BCL-2 expression, after examining the whole slide for the hot spots, and since no focal weak cytoplasmic positive cells were seen, the cases were divided into either positive or negative cases.

Total RNA extraction, purification, and quantification

From each sample, a serial of 15 sections of 5 µm were sliced. The first three sections were discarded to avoid contamination. Subsequently, sections were macro-dissected before RNA extraction and purification. Total RNA was isolated from FFPE tissues and purified with a spin column using the AmoyDx FFPE DNA/RNA Kit (AmoyDx, Xiamen, China). The detailed steps of extraction and purification were performed according to the manufacturer’s recommendations. Total RNA quantification was performed using QuantiFluor® RNA System (Promega, Madison, WI) and concentrations were measured using Quantus™ Fluorometer (Promega).

Dye-based one-step RT-qPCR

GoTaq® One-Step RT-qPCR (reverse transcription-quantitative polymerase chain reaction) system (Promega) was used to measure Axl receptor gene expression with beta-actin (ACTB) reference gene for relative gene expression analysis. The sequences of the primers used in this study are listed in Table 1.

**Table 1:** Primers' information. The Axl receptor primer was optimized at 65°C. Axl: anexelekto; ACTB: beta-actin.

Gene symbol	Accession number	Primer sequences	Temperature	Reference
Axl receptor	NM_021913	F: GTTTGGAGCTGTGATGGAAGGC	65°C	[16]
		R: CGCTTCACTCAGGAAATCCTCC		
ACTB	NM_001101.3	F: ATGTGGCCGAGGACTTTGATT	60°C	[17]
		R: AGTGGGGTGGCTTTTAGGATG		

The detailed steps, materials concentrations, and volumes were applied according to the manufacturer’s protocols. The final quantitative polymerase chain reaction (qPCR) was carried out in a total of 10 μL of reaction volume (master mix = 5 μL, forward primer = 0.5 μL, reverse primer = 0.5 μL, nuclease-free water = 2.5 μL, MgCl₂ = 0.25 μL, RT mix = 0.25 μL, and RNA template = 1 μL). RT-qPCR was carried out using a Mic-qPCR cycler (BMS, Upper Coomera, Australia). The cycling conditions at each step were as follows: complementary DNA (cDNA) synthesis (hold at 37°C for 15 minutes); reverse transcriptase enzyme deactivation and template denaturation (hold at 95°C for five minutes). After that, 40 cycles of a three-step cycling program were applied (95°C for 20 seconds, 60°C for 20 seconds acquiring on green for ACTB and 65°C for Axl, and 72°C for 20 seconds), with melt on green from 72°C to 95°C at 0.3°C/s. No template controls were included for each run.

Statistical analysis

Categorical variables, including age, tumor size (T), LN, ER, PR, HER2/neu, BCL-2, and Ki-67 immunoexpression binary statuses, were described using numbers and percentages and their significance in distribution was tested using Yate’s chi-square test and Fisher’s exact test. The effect size was described using Cramér's V and odd ratio (OR) run by RStudio (Posit, Boston, MA). Gene expression analysis was conducted using the relative gene expression method with the formula 2^-∆∆Ct^ [18] using the Microsoft Excel application (Microsoft Corporation, Redmond, WA). Numerical values of Axl receptor gene expression normality were checked using Q-Q plots, histograms, and the Shapiro-Wilk test, which all confirmed the non-normally distributed data suggesting the use of a non-parametric test (Mann-Whitney U test) to examine this receptor expression relationship with each categorical variable using SPSS version 25 (IBM Corp., Armonk, NY). The α threshold (0.05) was used to determine the significance of these tests.

## Results

Patients' clinicopathological data

The data were derived from the histopathological sheets and sorted in binary status. The results of the chi-square test with Yate's continuity correction results are demonstrated in Table 2, showing no significance regarding the tumor grade with age, size, and LN involvement. The majority of the studied population constituted of invasive ductal carcinoma (IDC) type (94%), accompanied by only 4% of infiltrative lobular type, and only one case (2%) presented IDC combined with lobular carcinoma (LC).

**Table 2:** Clinicopathological variables' correlation with the grade of the tumor. P-values are based on Yate's chi-square results. H.: high grade; L.: low/intermediate grade; T: tumor size; LN: lymph nodes.

	H. group		L. group		Total		P-value
	No.	%	No.	%	No.	%	
Above 50 years	10	20	9	18	19	38	1
Below 50 years	15	30	16	32	31	62	
T1	4	8	6	12	10	20	0.34
T2	9	18	13	26	22	44	
T3	11	22	5	10	16	32	
T4	1	2	1	2	2	4	
LN involved	16	32	10	20	26	52	1
LN not involved	9	18	15	30	24	48	

Hormone receptors and HER2/neu data analysis

Data regarding ER, PR, and HER2/neu were collected and extracted from patients’ information. Data were sorted in binary status (negative/positive). HER2/neu +3 was considered group 1 and HER2/neu negative, +1, and +2 were considered group 2. Table 3 demonstrates a comprehensive analysis of these markers with patient groups.

**Table 3:** HR and HER2/neu data analysis. P-values are based on Yate's chi-square results. H.: high grade; L.: low/intermediate grade; ER: estrogen receptor; PR: progesterone; HER2: human epidermal growth factor receptor 2.

	H. group		L. group		Total		P-value
	No.	%	No.	%	No.	%	
ER positive	12	24	22	44	34	68	0.006
ER negative	13	26	3	6	16	32	
PR positive	10	20	20	40	30	60	0.009
PR negative	15	30	5	10	20	40	
HER2 group 1	11	22	7	14	18	36	0.24
HER2 group2	14	28	18	36	32	64	

Fisher’s exact test showed either approximation or the exact p-value as the chi-square test. The p-values of both ER and PR status were less than 0.05, which indicates a significant association between these markers and the tumor grade, with Cramér's V of approximately 0.4 for both suggesting moderate association, and OR of 0.126 for ER and 0.167 for PR indicating that it is more likely for the L. group to be ER/PR+ and for the H. group to be ER/PR-. No significant association between HER2/neu status and tumor was observed. Additionally, these markers showed significant association with each other with p-values (ER/PR <0.0001 and Cramér's V of 0.8 describing a very large association, ER/HER2 of 0.02 and Cramér's V of 0.3 indicates moderate association, PR/HER2 of 0.002 and Cramér's V of 0.4, which also shows moderate association).

Biological subtypes resulted from the combination of HR status and HER2/neu status, according to the American Society of Clinical Oncology/College of American Pathologists (ASCO/CAP) guidelines [19]. For all cases, luminal A was the most dominant subtype constituting 48%, while luminal B and HER2-enriched subtypes both accounted for 20% with a difference in distribution between the groups, and triple-negative BC was present only in the H. group, which formed only 12% of the studied population. The p-value of 0.014 suggests a significant association between the graded groups in terms of the biological subtypes (accepting H_A_) and Cramér's V of 0.5 describes a strong association.

Immunohistochemistry for Ki-67 and BCL-2

**Figure 1:**
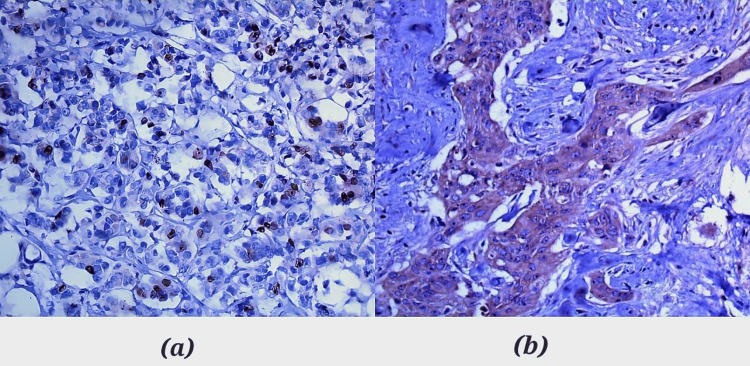
(a) Ki-67 nuclear expression on 20x with 30% expression in a high-grade specimen. (b) BCL-2 cytoplasmic expression on 20x showing positive staining in an intermediate-grade specimen. Images were taken using Leica DFC450 (Leica, Wetzlar, Germany) and LAS V4.5 (Leica Application Suite Version 4.5). Ki-67: Kiel 67; BCL-2: B-cell lymphoma 2.

**Figure 2:**
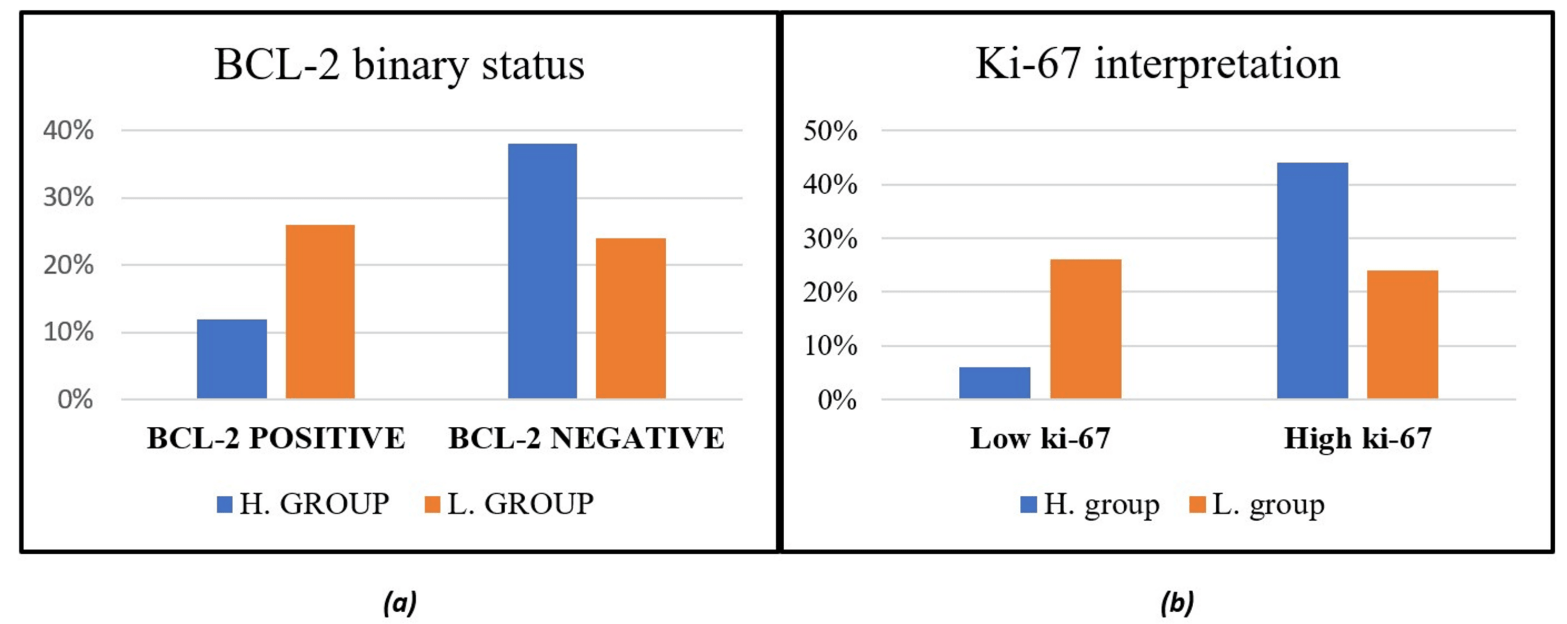
(a) BCL-2 binary status throughout groups. The L. group bars showed an increase in the positive cases (26%) and a decrease in the negative cases (24%) over the H. group bars, which accounted for 12% of positive cases and 38% of negative cases. (b) Ki-67 expression throughout groups. The H. group bars indicated higher percentage of patients with high expression (44%) than the L. group (24%); on the contrary, the percentage of patients with low expression in the L. group (26%) was approximately five times higher than the H. group (6%). The Y-axis presents percentages and the X-axis presents the binary categories as negative/positive. Ki-67: Kiel 67; BCL-2: B-cell lymphoma 2; H.: high grade; L.: low/intermediate grade.

Positive Ki-67 immunostaining was detected as brown nuclear staining in BC tumor cells (membrane staining and/or cytoplasmic was excluded from scoring), as shown in Figure 1. The results were described using percentages. A cut-off point of 20% was used to determine high (≥20%) and low (<20%) expression [20]. In regard to Ki-67 expression, 32% of the total cases had Ki-67 low expression (less than 20%), and 68% had high expression across the graded groups. As for BCL-2, positive immunostaining was detected as brown cytoplasmic staining in BC tumor cells, as shown in Figure 1. BCL-2-negative cases accounted for 62% of the total cases, while BCL-2-positive cases accounted for 38%. The results are demonstrated in Figure 2. As for stained benign cases, all resulted negative for both types, i.e., the non-neoplastic inflammatory cases and the fibroadenoma cases. Correlating these variables resulted in an X^2^ of 0.07 and a p-value of 0.79, which indicated an insignificant association between them.

BCL-2 comprehensive analyses

BCL-2 was correlated with the clinicopathological data, HR, and HER2, as demonstrated in Table 4.

**Table 4:** BCL-2 comprehensive analysis. P-values are based on Yate's chi-square results. BCL-2: B-cell lymphoma 2; H.: high grade; L.: low/intermediate grade; T: tumor size; LN: lymph nodes; ER: estrogen receptor; PR: progesterone receptor; HER2/neu: human epidermal growth factor receptor 2.

	BCL-2 positive		BCL-2 negative		Total		P-value
	No.	%	No.	%	No.	%	
Above 50 years	3	6	16	32	19	38	0.026
Below 50 years	16	32	15	30	31	62	
T1	4	8	6	12	10	20	0.261
T2	11	22	11	22	22	44	
T3	3	6	13	26	16	32	
T4	1	2	1	2	2	4	
H. group	6	12	19	38	25	50	0.08
L. group	13	26	12	24	25	50	
LN involved	10	20	16	32	26	52	1
LN not involved	9	18	15	30	24	48	
ER+	14	28	20	40	34	68	0.717
ER-	5	10	11	22	16	32	
PR+	12	24	18	36	30	60	0.953
PR-	7	14	13	26	20	40	
HER2/neu+	8	16	10	20	18	36	0.461
HER2/neu-	11	22	21	42	32	64	

Based on the calculated p-values, BCL-2 had a significant relationship only with the age groups, while it was insignificant when correlated with tumor size, LN involvement, the graded groups, ER, PR, and HER2. Tumor size correlation was also conducted using Fisher’s exact test and resulted in a p-value of 0.205, supporting the X^2^ results.

Ki-67 comprehensive analyses

Ki-67 was also correlated with the clinicopathological data, HR, and HER2, as demonstrated in Table 5.

**Table 5:** Ki-67 comprehensive analysis. P-values are based on Yate's chi-square results. Ki-67: Kiel 67; H.: high grade; L.: low/intermediate grade; T: tumor size; LN: lymph nodes; ER: estrogen receptor; PR: progesterone receptor; HER2/neu: human epidermal growth factor receptor 2.

	High Ki-67		Low Ki-67		Total		P-value
	No.	%	No.	%	No.	%	
Above 50 years	11	22	8	16	19	38	0.375
Below 50 years	23	46	8	16	31	62	
T1	5	10	5	10	10	20	0.425
T2	15	30	7	14	22	44	
T3	12	24	4	8	16	32	
T4	2	4	0	0	2	4	
H. group	22	44	3	6	25	50	0.006
L. group	12	24	13	26	25	50	
LN involved	20	40	6	12	26	52	0.057
LN not involved	14	28	15	30	29	58	
ER+	20	40	14	28	34	68	0.087
ER-	14	28	2	4	16	32	
PR+	16	32	14	28	30	60	0.016
PR-	18	36	2	4	20	40	
HER2/neu+	16	32	2	4	18	36	0.039
HER2/neu-	18	36	14	28	32	64	

Ki-67 was significantly associated with the grade of the tumor, PR, and HER2/neu. While it was not associated with the other variables. Fisher’s exact test supported X^2 ^results in regard to tumor size.

Axl receptor gene expression and comprehensive analyses

**Figure 3:**
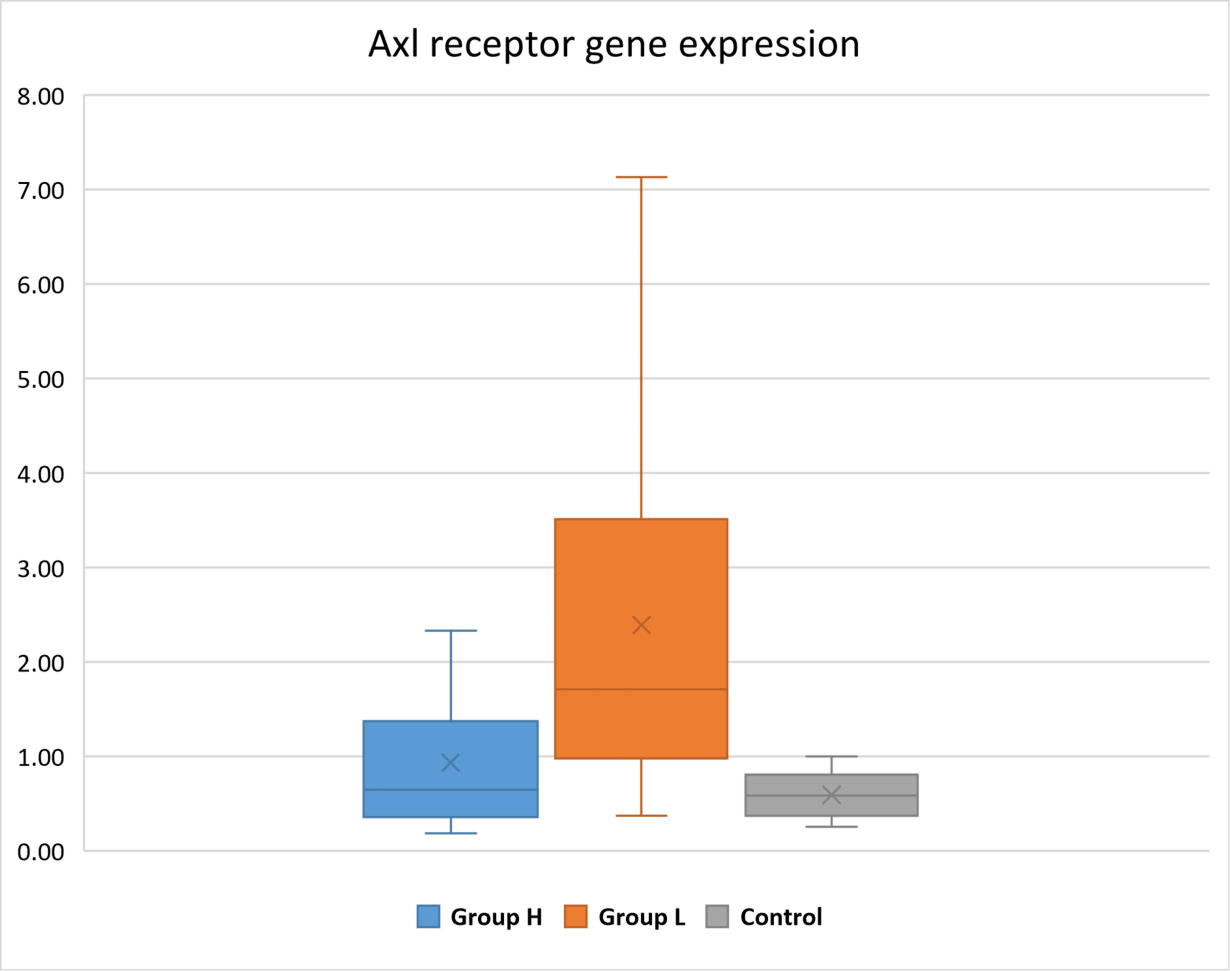
Axl expression average for the three groups. The H. group shows a higher value of expression in comparison with the control group, while the L. group demonstrates the highest values with the highest variability in expression within the individuals. The Y-axis represents the average fold change in the expression of the Axl receptor. H.: high grade; L.: low/intermediate grade; Axl: anexelekto.

Measuring Axl gene expression at the level of transcription was achieved by evaluating the folding level of mRNA transcripts with RT-qPCR. The expression of Axl in the H. group ranged between 0.19 and 20.96, with a median of 1.22, mode of 0.57, and standard deviation of 5.95, describing high variability in the expression of individuals of this group. The L. group range was 0.37-82.83, with the presence of extreme values; a median of 1.84, mode of 1.2, and standard deviation of 15.7 indicates a much higher variability in Axl expression. The control (benign) group range was 0.26-12.61, with a standard deviation of 3.43, which indicates moderate variability. The L. group average fold change in expression was higher than the H. group and the control group, as shown in Figure 3. The average fold change of expression for the three groups showed that the H. group was 1.74 times higher than the control group and the L. group was 3.74 times higher than the control group. The groups were correlated with each other and resulted in no significant difference between the H. and L. groups and H. and C. groups, while L. and C. groups were correlated and indicated that there was a significant difference between them with a p-value of 0.006. Also, the resulting p-values listed in Table 6 suggest that there is no significant association between the Axl receptor gene expression and other variables.

**Table 6:** Comprehensive analysis of Axl receptor expression with the study variables. The subtypes were divided into luminal and non-luminal. LN: lymph nodes; ER: estrogen receptor; PR: progesterone receptor; HER2/neu: human epidermal growth factor receptor 2; Ki-67: Kiel 67; BCL-2: B-cell lymphoma 2.

	Mann-Whitney U test	Wilcoxon W	Significance (2-tailed)
Age	285.5	475	0.857
LN involvement	309.5	609.5	0.961
ER	257.5	852.5	0.763
PR	278.5	488.5	0.670
HER2/neu	284	812	0.936
Ki-67	237	832	0.467
BCL-2	293.5	483.5	0.984
Subtype	257.5	852.5	0.763

## Discussion

In the current study, samples were collected primarily based on tumor grade. However, the attempt to be more inclusive with BC types was substantial in understanding the variability and heterogeneity of BC. The IDC type was dominant, accounting for 94% of the total samples collected, with little presence of LC. The mean age of 45.5 showed a close similarity with the population in the timeframe between 2007 and 2012, with a mean age of 46.8, which is relatively low [21]. The frequency of low grade was 0.06 within the studied population, which is comparatively low and it does not account for delays in diagnosis leading to progression, as Simpson et al. explained in 2005 that low- and high-grade BC follow distinct oncogenesis pathways [22]. A tumor size of 2-5 cm was the most prevalent, observed in 44% of cases, while tumors larger than 5 cm accounted for 32%, with only two cases involving the skin (T4b). Tumors less than 2 cm in size were present in 20% of cases. Additionally, nearly half of the cases had LN metastasis, while the other half did not. The presence of ER/PR+ was significantly high (58%) and associated with the L. group (40%), indicating a better prognosis. While HER2/neu did not show an association with the grade of the tumor, it was moderately associated with ER/PR markers. Luminal subtypes accounted for 68% (luminal A (48%) and luminal B (20%)), followed by HER2 enriched (20%) and triple-negative BC (12%) demonstrating differences with Baghdad’s subtypes ranking of luminal types (46.67%), followed by triple-negative (28.89%) and HER2 enriched (8.89%) [23].

Numerous studies examined the clinical outcomes associated with BCL-2, and its positive expression had a strong prognostic effect independent of the Nottingham Prognostic Index [24]. Two meta-analysis studies confirmed these findings; one included 18 studies with 2,285 cases, and the other included five studies with 11,212 cases. A conclusion was reached that “BCL2 was an independent indicator of favorable prognosis for all types of early-stage BC” [25,26]. BCL-2 anti-apoptotic protein was expressed in 38% and more likely in the age group below 50 years, which is less than what was stated in the previous study (52.8%) [27]. Based on our results, BCL-2 did not show any association with ER, PR, and HER2, and its expression correlation demonstrated that BCL-2 was independent of grade, subtype, tumor size, and LN and its prognostic effects require further investigation.

Tissue expression of Ki-67 complements prognostic information obtained from classical prognostic features such as tumor size, LN involvement, and pathological grading [28]. Haroon et al. [29] stated that biological features of BC are largely determined by cellular proliferation, and it was concluded that there is a strong correlation between the grade of the tumor and Ki-67 expression, which supports our findings of significant association with a p-value of 0.006 suggesting an association with higher nuclear grade. Additionally, high Ki-67 was significantly associated with HER2 status, which was consistent with Ragab et al. [28]. Moreover, Ki-67 expression showed a significant association with PR but did not correlate with ER.

Axl gene expression did not correlate with age, tumor size, LN involvement, ER, PR, HER2, Ki-67, BCL-2, and the biological subtype. Some of these findings were consistent with Jin et al. [9] and the other were not. These differences may be attributed to demographical and environmental variations or differences in measurement techniques. Using RT-qPCR provided precise expression value in the specimen being studied and indicated large variability in the results within the same groups with the presence of extreme values that skewed these statistical tests. In the current study, the gene expression results demonstrated that the Axl receptor was highly expressed in malignant tumors compared to the benign types, as was previously concluded by Jin et al. [9]. Our results also suggest that the Axl receptor was highly expressed in the L. group (low and intermediate grade), higher than the other groups, suggesting its association with better prognosis, which contradicted Adam-Artigues et al.'s findings [30].

In this study, obtaining a larger sample size was challenging due to time constraints and the limited variation in the availability of BC types within the studied population was also challenging.

## Conclusions

In this study, no significant association between the expression of BCL-2 and common tumor biology features was found, offering limited relevance for treatment decisions. The expression of Ki-67 emphasized its role as a valuable predictor of tumor behavior, potentially providing BC management approaches, and affirming its potential role in personalizing treatment plans. The higher expression of the Axl receptor in malignant tumors, especially in low/intermediate-grade tumors, highlighted its association with a favorable prognostic factor. In BC, the Axl receptor was primarily associated with epithelial-to-mesenchymal transition, invasion, migration, and cellular proliferation. However, no relationship between the Axl receptor, Ki-67 proliferation marker, and BCL-2 anti-apoptotic protein was found. These observations imply that this receptor activity may not be achieved by altering the proliferation or apoptotic mechanisms resulting in BC progression but by contributing to tumor aggressiveness through alternative mechanisms. Further research with a larger sample size is needed to confirm these findings and their implications for treatment. Given the numerous population differences, variations in epigenetics, and BC heterogeneity, it is important to address these gaps at both the transcriptomic and proteomic levels. By conducting this study, valuable insights into the classic prognostic markers and their associations in BC were observed, which may aid in refining BC management strategies by improving our understanding of these molecular markers.
